# The importance of sponges and mangroves in supporting fish communities on degraded coral reefs in Caribbean Panama

**DOI:** 10.7717/peerj.4455

**Published:** 2018-03-29

**Authors:** Janina Seemann, Alexandra Yingst, Rick D. Stuart-Smith, Graham J. Edgar, Andrew H. Altieri

**Affiliations:** 1 MarineGEO, Smithsonian Tropical Research Institute, Panamá, Republic of Panama; 2 University of Pittsburgh, Pittsburgh, PA, United States of America; 3 Institute for Marine and Antarctic Studies, University of Tasmania, Hobart, Tasmania, Australia; 4 Department of Environmental Engineering Sciences, Engineering School of Sustainable Infrastructure and Environment, University of Florida, United States of America

**Keywords:** Foundation species, Habitat connectivity, Nursery habitat, Overfishing, Sponges, Reef degradation, Trophic imbalance

## Abstract

Fish communities associated with coral reefs worldwide are threatened by habitat degradation and overexploitation. We assessed coral reefs, mangrove fringes, and seagrass meadows on the Caribbean coast of Panama to explore the influences of their proximity to one another, habitat cover, and environmental characteristics in sustaining biomass, species richness and trophic structure of fish communities in a degraded tropical ecosystem. We found 94% of all fish across all habitat types were of small body size (≤10 cm), with communities dominated by fishes that usually live in habitats of low complexity, such as Pomacentridae (damselfishes) and Gobiidae (gobies). Total fish biomass was very low, with the trend of small fishes from low trophic levels over-represented, and top predators under-represented, relative to coral reefs elsewhere in the Caribbean. For example, herbivorous fishes comprised 27% of total fish biomass in Panama relative to 10% in the wider Caribbean, and the small parrotfish *Scarus iseri* comprised 72% of the parrotfish biomass. We found evidence that non-coral biogenic habitats support reef-associated fish communities. In particular, the abundance of sponges on a given reef and proximity of mangroves were found to be important positive correlates of reef fish species richness, biomass, abundance and trophic structure. Our study indicates that a diverse fish community can persist on degraded coral reefs, and that the availability and arrangement within the seascape of other habitat-forming organisms, including sponges and mangroves, is critical to the maintenance of functional processes in such ecosystems.

## Introduction

Coral reef fishes are useful model communities for exploring drivers of species diversity at landscape and regional scales (Galzin et al., 1994; Fabricius et al., 2005; Knowlton et al., 2010; Wilson et al., 2010). They are sensitive to changes in habitat and anthropogenic impacts—a particular concern given that fishes play an important role in coral reef ecosystems, and declines of coral reef fishes threaten people’s livelihoods and food security (Cesar, 2000; Cesar, Burke & Pet-Soede, 2003; Bellwood et al., 2004; Paddack et al., 2009). A variety of human impacts are responsible for coastal degradation, including habitat destruction, eutrophication, and sedimentation (Hughes, 1994; Jackson et al., 2001; Aronson et al., 2003). Climate change has contributed to ecosystem decline through coral die-off from bleaching, hypoxia events and storms (Wilson, 2006; Alvarez-Filip et al., 2009; Wilson et al., 2010; Altieri et al., 2017). The consequences of these events are structural collapses and habitat homogenization in coral reefs, effects which have a variety of potential direct and indirect implications for resident organisms (Bell & Galzin, 1984; Jackson et al., 2001; Kuffner et al., 2007; Wilson et al., 2010; Alevizon & Porter, 2015; Mora, 2015).

Additional factors contributing to declining reef fish abundances are unsustainable fisheries and increasing demand for fish products for a growing human population (Hodgson, 1999; Jackson et al., 2001; Zaneveld et al., 2016). The overexploitation and disproportionate targeting of large size classes and high trophic levels affect fish population structure, growth, and reproduction, and contribute to a trophic imbalance and shifts in trait composition in the reef fish community (Hixon, Johnson & Sogard, 2014). This in turn has led to further changes in habitat structure, phase shifts from coral to algal communities, and decreasing ecosystem stability (Saila, Kocic & McManus, 1993; Jennings & Lock, 1996; White & Jentsch, 2001).

Reef fish populations have also been negatively affected by the loss of coastal habitats that provide important nurseries (Nagelkerken et al., 2000). The nursery-role concept suggests that many reef fishes (e.g., families Lutjanidae, snappers; Serranidae, groupers; Haemulidae, grunts) have life cycles that include seagrass meadows and mangroves as nursery and feeding grounds (Beck et al., 2001; Nagelkerken et al., 2002; Unsworth et al., 2008; Ley, 2014; Serafy et al., 2015). Seagrass meadows and mangrove forests have high primary and secondary productivity relative to unvegetated substrates, and support high diversity and abundances of reef fishes (Nagelkerken et al., 2000; Beck et al., 2001; Mumby et al., 2004). Many fish species on coral reefs therefore depend on the connectivity to, and integrity of, associated habitats.

Our study region, the Caribbean Sea, has experienced declining reef fish populations as a result of pollution, ecosystem degradation and unsustainable reef fisheries (Hughes, 1994; Gardner et al., 2003; Bellwood et al., 2004; Paddack et al., 2009). These problems appear particularly prominent at our focal study area in Bocas del Toro on the Caribbean coast of Panama, where rapid human population growth connected with agriculture (banana industry) and tourism has accelerated the decline of water quality and the physical destruction of reefs, and has increased fishing pressure (Guzmán & Jiménez, 1992; Collin, 2005; D’Croz, Rosario & Gondola, 2005; Cramer, 2013; Aronson et al., 2014; Seemann et al., 2014). Bocas del Toro encompasses a coastal coral reef-seagrass-mangrove system in a semi-enclosed lagoon. It is composed of six major islands and the mainland that surround the Almirante Bay, and includes mangroves fringing the mainland and mangrove islands scattered across the bay (Collin, 2005; Guzmán et al., 2005). Reefs are typically dominated by corals with a high stress tolerance, including *Porites furcata* in shallow (1–4 m) and *Agaricia* spp. (>3 m) in the deeper areas (Seemann, 2013; Aronson et al., 2014; Seemann et al., 2014). Associated seagrass meadows are dominated by *Thalassia testudinum* (turtlegrass). Mangrove fringes are comprised of *Rhizophora mangle* (red mangrove). Several rivers, creeks and oceanic inlets discharge sediments and nutrients into the bay (Beulig, 1999; Collin, 2005). Bleaching and low oxygen events occur regularly due to lagoonal characteristics including retention of warm water and depletion of oxygen (Kaufmann & Thompson, 2005; Seemann et al., 2014; Altieri et al., 2017). Bocas del Toro reefs potentially represent a model system for improving predictions relevant throughout the region due to their exposure to common stressors, such as high terrigenous runoff, nutrient levels, and overfishing, that are afflicting other coral reefs in the Caribbean (Riegl et al., 2009; Sammarco & Strychar, 2009; Leinfelder et al., 2012; Aronson et al., 2014).

This study aims to characterize the ecosystem attributes that facilitate the maintenance of essential functions, biodiversity and biomass of coral reef fish communities in a degraded ecosystem. Specifically, we (1) quantify the fish community at 67 sites in five bioregions of the Caribbean to assess the status of our focal study system in Bocas del Toro along a gradient of ecosystem degradation and over-fishing, (2) examine the effects of proximity of mangroves and seagrass for fish communities on coral reefs, and (3) identify characteristics of coral reef habitat that are positively related to biomass, abundance and structure of the fish community. Addressing these objectives contributes to a better understanding of how landscape-scale features underlie the resilience of degraded coastal habitats.

## Methods

### Study system

To place results within the wider regional context, we conducted fish surveys at reefs with different fishery management restrictions in different Caribbean ecoregions.

**Figure 1:**
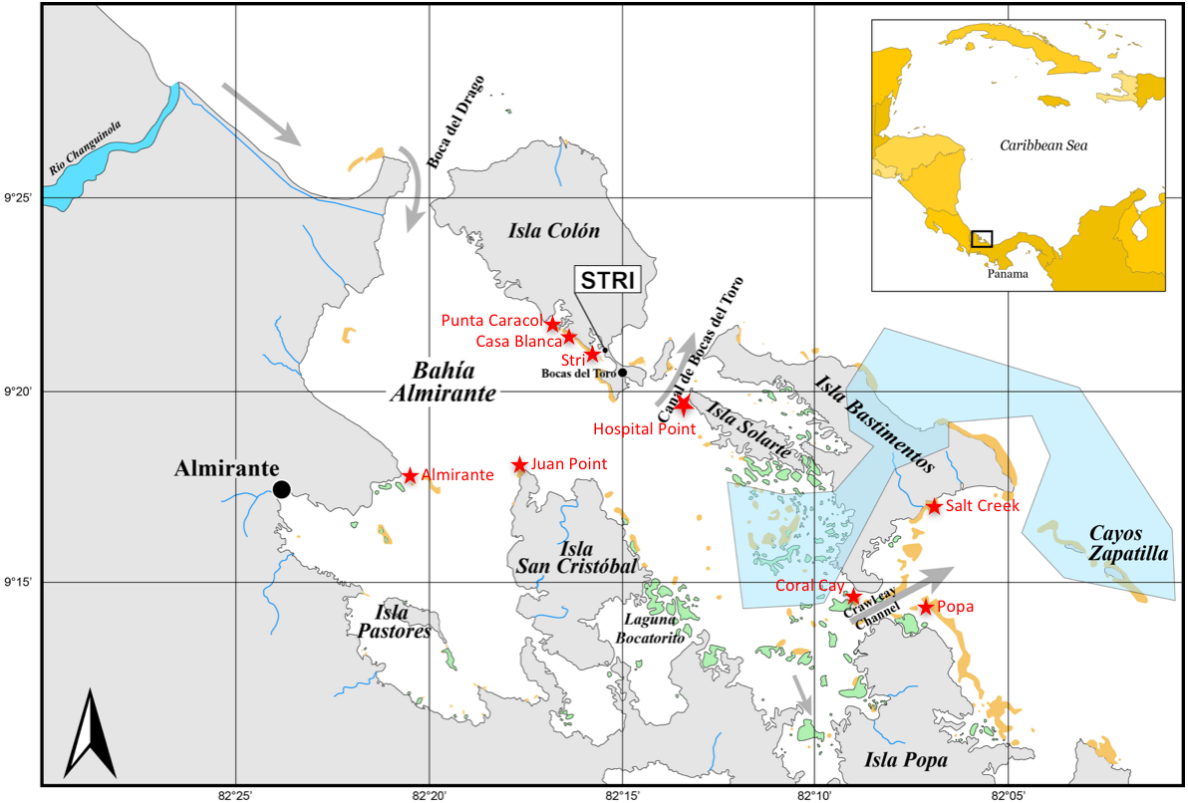
Sampling sites in Bocas del Toro. Three reef sites (Punta Caracol, Casa Blanca, Almirante) possess close connectivity with mangrove habitat (within 100 m), three sites (STRI, Juan Point, Coral Cay) represent reef sites further away from mangroves (100–250 m), and three reef sites (Popa, Salt Creek, Hospital Point) are not closely connected to mangroves (>750 m). Yellow areas are reefs and green areas are mangrove islands, gray is land, white is ocean, blue is river and blue polygon is a poorly enforced MPA.

In our focal study areas of Bocas del Toro on the Caribbean coast of Panama (Fig. 1), we also conducted comprehensive surveys of fish communities, benthic surveys, and water quality measurements in adjacent seagrass and mangrove fringe areas. All data from Bocas del Toro were collected from May to July 2015. Data for the other Caribbean regions were collected from 2012 to 2015. Research was conducted under a Scientific Permit from the Ministry of the Environment Panama (MiAmbiente) and Autoridad de los Recursos Acuáticos de Panamá (ARAP) with the Number: SE/APO-1-15 & 10b.

### Caribbean data set

#### Fish surveys

We conducted visual fish surveys using the Reef Life Survey (RLS) method 1 protocol (Edgar & Stuart-Smith, 2014) at 67 reef sites in the following five ecoregions (Spalding et al., 2007): Southern Caribbean (14 sites, Bonaire), Southwestern Caribbean (31 sites, Bocas del Toro, Kuna Yala, Archipelago of San Andres), Greater Antilles (one site, Grand Cayman), Floridian (17 sites, Florida Keys) and Bahamian (four sites, Turks and Caicos Islands). Surveys involved underwater visual censuses by scuba divers at reef sites (each with 2–6 replicate transects) in depths of 1 to 35 m. Divers counted and assigned all fish species observed within binned size-classes along a 50 × 5 m belt transect (250 m^2^). All fishes sighted on each transect were recorded on a waterproof datasheet as the diver swam along the transect at approximately 2 m min^−1^. We identified fish species to the highest taxonomic resolution possible, and estimated body length for each individual. The order of priority for recording accurately was to first ensure all species observed along transects were included, then individuals of larger or rare species were accurately counted, then estimates were made of abundances of common species. If an individual could not be identified underwater, a photograph was taken for later identification. Abundance, size and species identity were used to estimate biomass in kg ha^−1^ using conversion factors provided by Fishbase (http://www.fishbase.com), as described by Edgar & Stuart-Smith (2014).

### Bocas del Toro data set

#### Fish surveys

The RLS method employed on coral reefs in five ecoregions was also applied in seagrass and mangrove habitats located within 250 m of reef sites in Bocas del Toro. Seagrass sites ranged in depth from 1 m to 4 m, whereas mangrove fringe root systems had maximal depth of 2 m. Mangrove surveys were conducted amongst the mangroves prop roots below the upper intertidal fringe with counts and size estimates made for all fishes in a 5 m wide belt within the mangrove root system. Two 50 m transects were laid end-to-end along the mangrove fringe given that side-by-side replicate transects typical of the RLS protocol could not be applied within mangrove root habitats.

#### Habitat assessment

We conducted benthic surveys to characterize coral reef and seagrass bed habitats. Reef and seagrass benthos were analyzed with 20 photo quadrats (0.5 m^2^), which were taken every 2.5 m along the 50 m long transects at each site. Photos were analyzed via point counting using the Coralnet annotation tool (coralnet.ucsd.edu). A total of 25 points were randomly distributed on each photo and categorized. Substratum categories for analyses were: healthy hard coral, bleached hard coral, recently-dead coral, anemones, non-calcifying corals (including hexacorals and octocorals), sponges, sessile worms (tube worms, mostly polychaetes), zoanthids, rubble, sand, rock, calcifying algae, seagrass and macroalgae. If sessile organisms were too small for identification or obscured by dark shadows, then they were excluded from the dataset. In addition, the distance between reef sites surveyed and nearest mangrove was measured using GPS coordinates (Table 1).

**Table 1:** Major habitat characteristics and location of monitoring sites. Sites 7, 8 and 9 did not have mangroves in close proximity (≤250 m); site 9 also did not have a seagrass meadow close to the reef, effective juvenile habitats were calculated from the fish abundance in mangroves (w/o small bodied fish) compared to reef fish abundance.

	Site	Coordinates Lat	Coordinates Long	Depth reef (m)	Depth seagrass (m)	Distance Reef-mangrove (m)	Sponge cover %	Live hard coral cover %	Hard substrate %	Reef fish Biomass kg ha^−1^	Seagrass fish Biomass kg ha^−1^	Mangrove fish Biomass kg ha^−1^	Seagrass as effective juvenile habitats%	Mangroves as effective juvenile habitats%	Reef fish abundance ha^−1^	Seagrass fish abundance ha^−1^	Mangrove fish abundance ha^−1^	Reef fish richness	Seagrass fish richness	Mangrove fish Richness
1	Punta Caracol	9.3757°	−82.2997°	3	2	65	9.5	41.5	33	201	25	111	109	131	12,929	2,820	17,423	38	12	21
2	Casa Blanca	9.3588°	−82.2737°	3	1	70	17.5	2.5	71	67	32	47	102	73	18,741	67,660	17,570	30	9	16
3	Almirante	9.2900°	−82.3429°	3	2	90	19.5	36.5	71	206	2	202	13	59	11,105	2,560	1,202,510	28	6	15
4	STRI Point	9.3483°	−82.2625°	3	4	120	19.5	3.0	57	257	24	15	49	29	71,076	73,153	42,390	35	15	19
5	Juan Point	9.3003°	−82.2921°	4	1	170	17.6	46.4	69	94	14	32	86	29	24,045	31,760	200,660	30	11	10
6	Coral Cay	9.2435°	−82.1478°	5	2	230	2.0	16.0	51	25	12	11	54	139	1,717	50,850	42,060	25	7	9
7	Popa	9.2336°	−82.1120°	3	1	700	1.1	26.9	61	60	2		23		2,608	560	17,423	24	9	
8	Salt Creek	9.2815°	−82.1012°	6	2	950	0.5	24.8	99	13	0		105		1,688	1,290		15	12	
9	Hospital Point	9.3326°	−82.2220°	5.5		900	0.5	96.0	33	12					1,946			16		

#### Water quality measurements

Water quality was assessed by quantifying temperature (°C), salinity (psu), water depth (m), total dissolved solids (TDS, mg L^−1^), dissolved oxygen (mg L^−1^), pH, turbidity (FNU), chlorophyll (µg L^−1^), blue–green algae concentrations (µg L^−1^), and dissolved organic matter (fDOM, RFU) with an Exo2 multiparameter sonde (Xylem brand; YSI, Yellow Springs, OH, USA). The sonde was positioned ∼10 cm above the bottom in each habitat (reef, seagrass and mangrove fringe). Measurements were recorded at intervals of 1–6 min over a time period of at least 30 min during the fish surveys, and constrained to the mid-day hours 10 am–3 pm, hence measurements were subject to the daily variability of weather conditions or tidal cycles.

### Data analyses

The Caribbean reef fish data set was used to characterize the fish community in relation to the protection status of the sites. All 67 sites from the five different ecoregions were individually classed by management type using the criteria of Edgar & Stuart-Smith (2014): NTZ (no take zones, *n* = 27), RZ (restricted zones that allow some methods of fishing within a MPA, *n* = 19) and OZ (open zones where fishing is unrestricted, *n* = 12). These data were compared to data from Bocas del Toro (OZ, *n* = 9). Replicated surveys from each site were averaged.

Data from the fish surveys were used to calculate fish community metrics, including total abundance (density), abundance of major fish families within size bins (≤10 cm; >10–20 cm; >20 cm), total biomass, biomass of fishes ≤10 cm, and total species richness. We also calculated the mean trophic level as an abundance weighted mean of the reef fish community by multiplying the trophic level of each species by their log abundance, summing these values across species recorded on a transect, and dividing by the total log abundance of all fishes on the transect. The classification of the trophic level (2–5) for each species was based on feeding strategy: herbivores and detritivores (2–2.1), omnivores (2.2–2.7), low-level carnivores (2.8–3.4), mid-level carnivores (3.5–3.9) and high-level carnivores (4–4.5) (classification and values obtained from Fishbase; http://www.fishbase.org). We also compared preferred substrate types and resilience factors of the fish species (values obtained from Fishbase), the latter estimated from population doubling time (low, medium, high). Fish community metrics were averaged within sites and compared among regions for significant differences using one-way ANOVA or a Student’s *t*-test.

For the Bocas del Toro dataset only, we assessed whether mangroves and seagrasses provided juvenile or alternative habitats to coral reefs by comparing the abundance (log transformed) and composition of fishes in the different habitat types. We assumed that higher abundances of fishes amongst mangroves and seagrasses compared to reefs, and high species similarity, indicates higher likelihood and magnitude of migration and exchange rates. We excluded small-bodied species (maximum total length ≤12.5 cm), which are presumably non-migratory fish species (Dahlgren et al., 2006), such as *Apogon townsendi* (belted cardinalfish), *Coryphopterus* spp., and *Canthigaster rostrata* (caribbean sharpnose-puffer). A principal component analysis (PCA) on correlations (fish abundance log transformed, only fish >12.5 cm) was used to compare differences in the fish communities between reefs at different distances to mangroves and seagrass.

We also tested for correlations between environmental factors and the reef fish community metrics across all sites. Environmental factors included reef cover, cover of the seagrass benthos, distance to mangroves and water quality parameters. Fish metrics included species richness, biomass, size structure, the abundances of individual fish species, tropic levels, preferred substrate types and resilience factors. Data were characterized using a scatterplot matrix (see Supplemental Information 4) and nonparametric Spearman’s tests for pairwise correlation probabilities. For all statistical analyses, fish abundance data were log-transformed to down-weight the extremely high abundance of a few fish species (Edgar et al., 2014). All statistical analyses were conducted using JMP Software 13.01.

## Results

### Characteristics of the fish community

**Figure 2:**
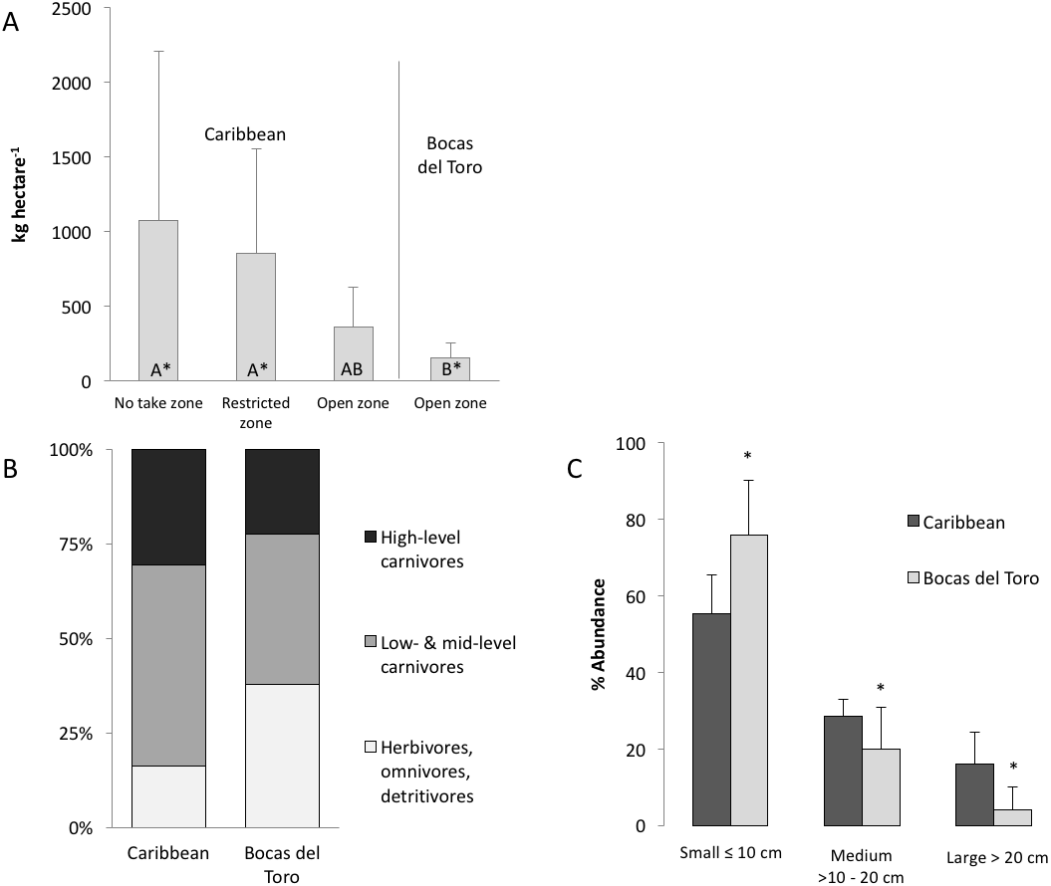
Biomass and composition of the fish community in the Caribbean and Bocas del Toro. (A) The comparison of the total biomass (AVR ± SD) from RLS conducted across the Caribbean, divided in no take zones, restricted zone and open zones, and open zones in Bocas del Toro, groups with different letters are significantly different. (B) Distribution of trophic guilds based on total biomass: high-level carnivores (trophic level 4–4.5), low and mid-level carnivore (trophic level 2.8–3.9), herbivores, omnivores and detrivores (trophic level 2–2.7). (C) The abundance of fish subdivided in size classes (AVR ± SD), which are indicative of fishing pressure (a skew towards smaller body size implies fishing). Asterisk represents significant differences between size abundance data from Bocas and the Caribbean.

We recorded a total of 77 fish species across all habitats in Bocas del Toro, of which 61 species were found on coral reefs. The average mean richness per transect was 29 ± 7 (SD) species. This value was low compared to our other Caribbean survey sites which had a mean richness per transect of 52 ± 4 species (with a cumulative total of 196 species recorded in the whole Caribbean) (Stuart-Smith et al., 2013; Edgar & Stuart-Smith, 2014). Fish biomass on Bocas del Toro reefs (71 ± 63 kg ha^−1^) was also lower than on other Caribbean reefs in both no-take zones and MPAs with restricted fishing (ANOVA, *P* = 0.02 and 0.001, respectively), although the difference was not significantly lower for open zones (*P* > 0.05) (Fig. 2A). Moreover, the range of total observed fish biomass in Bocas del Toro (30–1,350 kg ha^−1^) represents the lowest range found amongst fish surveys conducted in the Caribbean, which were 140–5,930 kg ha^−1^ elsewhere.

The biomass of herbivorous, omnivorous and detrivorous fishes in Bocas del Toro (trophic level 2–2.7) was 37% of the total biomass and 76% of all individual fishes counted (Fig. 2B). Herbivores alone comprised 27 ± 3.5% (SD) of biomass versus 10 ± 4% across the wider Caribbean. Pomacentridae (damselfishes) and Scarinae (parrotfishes) were the predominant taxa in terms of biomass. *Scarus iseri* (striped parrotfish) contributed 72% of the parrotfish biomass. High-level carnivores contributed 22 ± 3.5% of total fish biomass, versus 31 ± 4% elsewhere in the Caribbean. Dominant high-level carnivores in Bocas del Toro were *Caranx ruber* (bar jack), *Cephalopholis cruentata* (graysby), *Hyplopectrus nigricans* (black hamlet) and *Scomberomorus regalis* (cero). There was a trend for fish communities in Bocas del Toro to exhibit a greater proportion of total biomass comprised of herbivores, omnivores and detrivores (trophic level: 2–2.7), and a lower proportion comprised of high-level carnivores (trophic level: 4–4.5), relative to other Caribbean reefs, although the difference was not significantly different for either group (Fig. 2B).

A total of 94% of all fishes observed across all habitat types (reef, seagrass, mangrove) in Bocas del Toro were in the smallest size class (≤10 cm length). Fishes ≤10 cm represented 59% of the total biomass within the reefs. The abundance of fishes within the smallest size class (≤10 cm) was significantly higher in Bocas del Toro than other Caribbean reefs (ANOVA, *P* < 0.0001), whereas the abundances of medium- (>10–20 cm) and large- (≥20 cm) sized fishes were significantly lower (ANOVA, *P* < 0.0001) (Fig. 2C). This pattern was also evident when comparing Bocas del Toro with other Caribbean reefs across fish families (Table 2).

**Table 2:** Major fish families (only considering >10 counts ha^−1^ in average in one of the size bins).

	**Caribbean**			**Bocas del Toro**								
	**Reef**			Reef	Seagrass	Mangrove	Reef	Seagrass	Mangrove	Reef	Seagrass	Mangrove
	≤10 cm	>10–20 cm	>20 cm	≤10 cm	≤10 cm	≤10 cm	>10–20 cm	>10–20 cm	>10–20 cm	>20 cm	>20 cm	>20 cm
Acanthuridae	317	351	127	113	127	120	233	0	20	0	0	0
Balistidae	100	380	145	0	0	0	0	0	0	0	0	0
Carangidae	296	1,78	145	321	20	2,93	330	80	100	40	0	0
Clupeidae	11,500	0	0	0	46,000	278,080	0	0	0	0	0	0
Ephippidae	0	80	280	0	0	0	30	0	0	0	0	0
Gerreidae	0	30	20	0	600	155	0	0	20	0	0	0
Gobiidae	6,239	0	0	18,182	30	80	0	0	0	0	0	0
Grammatidae	434	0	0	0	0	0	0	0	0	0	0	0
Haemulidae	1,959	1,395	160	379	752	823	457	300	70	20	0	0
Holocentridae	253	441	50	0	0	0	20	0	0	0	0	0
Inermiidae	300	3,444	0	0	0	0	0	0	0	0	0	0
Kyphosidae	463	733	160	0	0	0	0	0	0	0	0	0
Labridae	1,749	659	96	254	568	580	252	180	80	0	0	0
Loliginidae	0	240	0	0	0	0	0	0	0	0	0	0
Lutjanidae	263	800	279	80	137	559	80	20	350	0	0	20
Mullidae	245	429	229	50	20	0	20	0	0	0	0	0
Pomacentridae	2,145	414	20	618	325	123	110	0	0	0	0	0
Scaridae	741	252	196	494	753	979	333	20	173	80	0	0
Sciaenidae	532	176	40	60	0	0	60	0	0	0	0	0
Serranidae	855	107	208	297	72	40	93	0	0	0	0	0
Sphyraenidae	120	2,100	180	0	0	40	0	20	40	0	0	100
Tetraodontidae	247	0	0	193	100	20	0	0	0	0	0	0

### Relationships between environmental factors and fish community composition

Some environmental parameter and habitat factors were associated with the reef fish community metrics in Bocas del Toro. Some environmental factors were not independent, as sponge cover was negatively correlated with the distance to mangroves and also positively to chl *a* (*R*^2^ = 0.60 and *R*^2^ = 0.70, respectively, *P* < 0.01). The other water quality parameters were not found to correlate with any fish community or species metrics. Sponge cover was the strongest positive correlate among all environmental parameters for species richness (*R*^2^ = 0.5, *P* < 0.01), small fish ≤10 cm biomass (*R*^2^ = 0.85, *P* < 0.01), and trophic level of the fish community (*R*^2^ = 0.89, *P* < 0.01). The abundance of *Abudefduf saxatilis* (sergeant major) was significantly correlated with sponge cover (*R*^2^ = 0.62, *p* = 0.0027). Survey sites characterized by high sponge cover and low distance to mangroves were characterized by fishes such as *Abudefduf saxatilis* (sergeant major), *Hypoplectrus nigricans* (black hamlet), *Coryphopterus personatus* (masked goby) and *Coryphopterus glaucofraenum* (bridled goby). *Scarus iseri* (striped parrotfish), *Stegastes partitus* (bicolor damselfish) and *Cephalopholis cruentatus* (graysby) had a positive association with recently dead corals, however, the cover of dead corals was negatively correlated with the abundance of most fish species.

**Figure 3:**
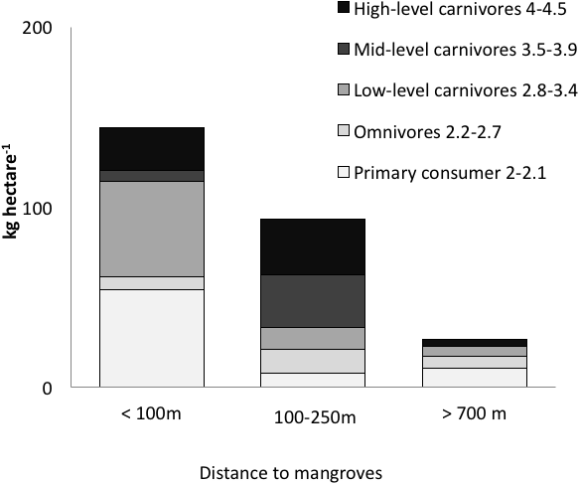
Biomass of trophic guilds of reef fish. Data were pooled by sites with a similar distance to mangroves: <100 m (Punta Caracol, Casa Blanca, Almirante), 100–250 m (STRI, Juan Point, Coral Cay) and >700 m (Popa, Salt Creek, Hospital Point) (see Table 1 for detail).

**Figure 4:**
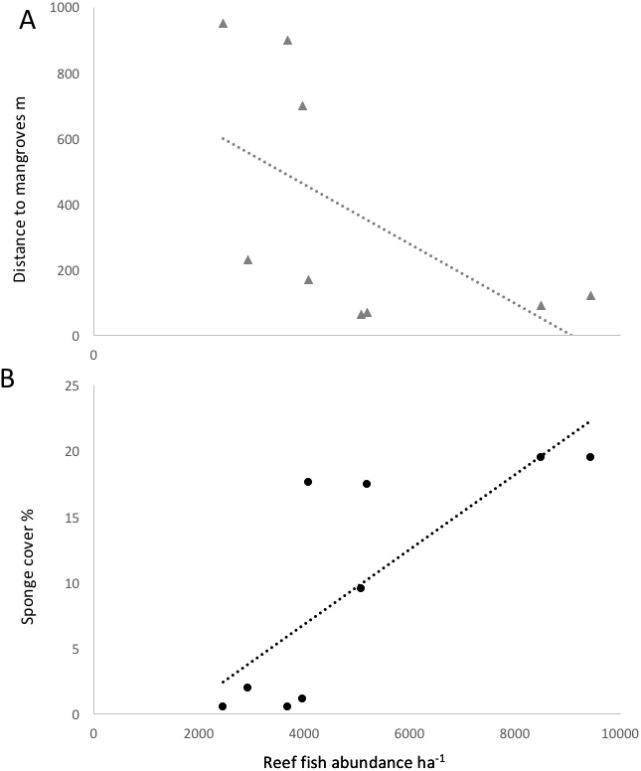
Correlation of reef fish abundance (w/o small-bodied fish) and (A) distance to mangroves or (B) sponge percent cover.

Fish species richness on a given reef was positively correlated with richness values in nearby mangroves (*R*^2^ = 0.76, *P* < 0.05). The three reef sites with low sponge cover and without mangroves in close proximity (Salt Creek, Popa, Hospital Point) showed lower biomass, abundances and species richness of fishes (Table 1, Figs. 3 and 4). The site without either mangroves or seagrass nearby (Hospital Point) showed the lowest species richness.

**Figure 5:**
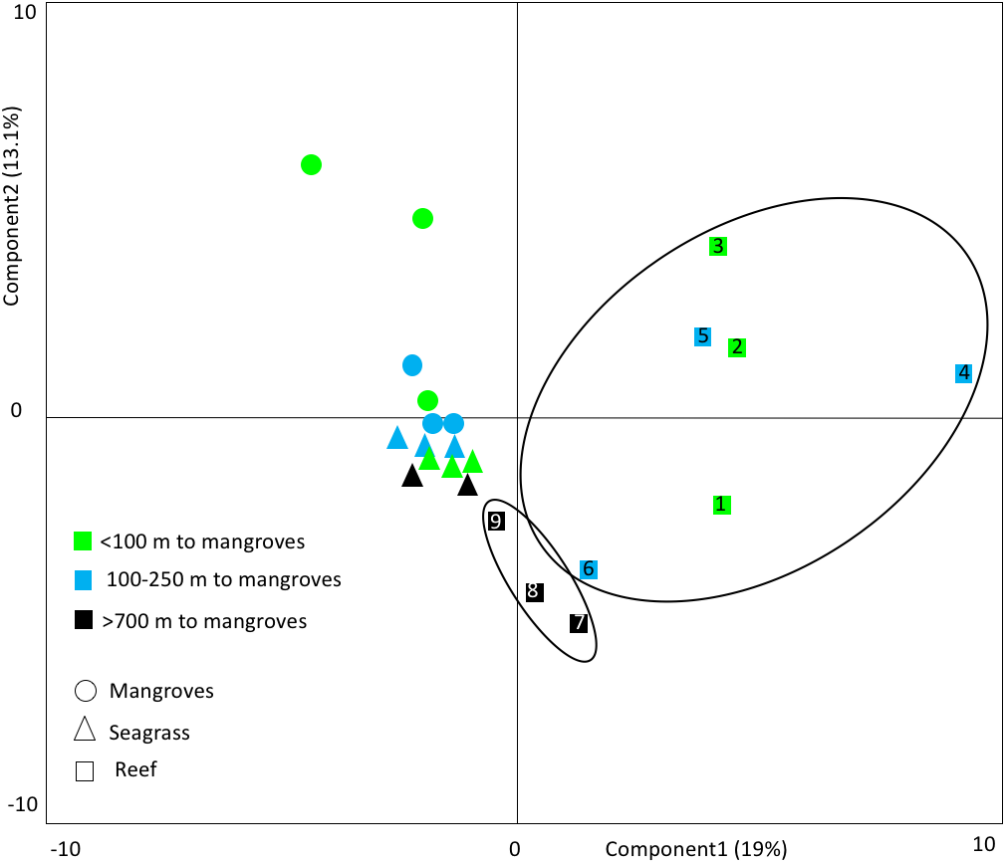
Principal component analyses. This PCA shows the clustering of fish communities, considering species composition and abundance (log transformed). Small bodied fish ≤12.5 cm was excluded. Reefs >700 m from mangroves cluster together as a group, separate from the reefs that are closer to mangroves. This indicates that mangrove distance has a strong influence on reef community types. The numbers refer to the site numbers in Table 1.

Distance to mangroves was also identified as a factor that influenced reef fish communities (Fig. 5), suggesting that proximity to mangroves has a strong influence on reef fish communities, likely by mangroves functioning as effective nursery grounds and as alternative complex habitats. The proportion of carnivorous fishes was higher at the sites closer to the mangroves than those sites that were further away (ANOVA, *P* < 0.01, Fig. 4). However, a more detailed look at the fish communities revealed that the sites at an intermediate distance from mangroves (STRI, Juan Point, Coral Cay) possessed a significantly higher proportion of top-level carnivores than sites that were closer or further away (Fig. 4, ANOVA, *P* < 0.01).

The highest abundances of all fish observed were recorded for the families Pomacentridae (damselfishes) and Gobiidae (gobies) (Table 2). However, Gobiidae were abundant only at sites close to mangroves. *Coryphopterus personatus* (masked goby) dominated these sites, with abundances up to 13 individuals m^−2^. RLS conducted elsewhere in the Caribbean (e.g., San Andres Archipelago, 350 km distant) revealed much lower densities for the same species (0.2 individuals m^−2^).

Generally, fishes with life cycles closely associated with hard corals (Lewis, 1997), such as Pomacanthidae (angelfishes), were present in very low numbers on the reefs of Bocas del Toro (<1 per transect). Other reef fishes typically associated with hard substrates with a high complexity, such as Balistidae (triggerfishes), Apogonidae (cardinalfishes), Muraenidae (moray eels), Sciaenidae (drums), Pseudochromidae (dottybacks) and Serranidae (grouper), were scarce within the bay (<1 per transect). Many fish species, including those at higher trophic levels, such as *Diodon hystrix* (porcupinefish), *Ginglymostoma cirratum* (nurse shark), *Gymnothorax Funebris* (moray eel), *Lutjanus jocu* (dog snapper), *Ocyurus chrysurus* (yellowtail snapper), *Pomacanthus arcuatus* (gray angelfish) were observed only on reefs with mangroves in close proximity (≤250 m distance).

## Discussion

Our surveys revealed that the fish fauna in Bocas del Toro is depauperate in species richness and biomass by Caribbean standards. We found evidence that the fish community is representative of a degraded and overexploited ecosystem, characterized by numerical dominance of fishes that are small bodied and also typical of habitats of low complexity, such as Pomacentridae and Gobiidae, with few representatives of fish families that achieve body sizes targeted by fisheries or that are commonly associated with high-relief coral reefs. Nevertheless, sponge cover and proximity to mangroves were found to be positively correlated with fish species richness, biomass, abundance and trophic level. This pattern suggests that sponges as habitat-forming reef organisms, and mangroves as nursery grounds and alternative habitats, continue to provide critical habitats for the reef fish communities in a degraded ecosystem, and therefore counteract some effects of reef degradation.

Some fishes appeared to be an indicator species for the overall trends observed at our study site. One example is the goby *Coryphopterus personatus,* which forms schools that hover in a vulnerable position above the bottom in extremely high abundances (65-fold higher abundances than in the San Andres Archipelago). Moreover, fish surveys in our Bocas del Toro study area from 2002 revealed densities an order of magnitude lower at 1.2 individuals m^−2^ (Dominici-Arosemena & Wolff, 2005). We suggest that this goby is an indicator species for overfished reefs that benefits from a loss of predatory fishes that historically limited their densities. Another indicator species is *Scarus iseri*, which is ecologically important given its role as the predominant herbivorous fish in Bocas del Toro (Kuempel & Altieri, 2017). This species likely plays an important role supporting the growth of sponges and corals by cropping competing macroalgae. Third, *Abudefduf saxatilis* was identified as an indicator for sponge cover, which in turn is a factor positively correlated to fish richness, biomass, abundance and relatively high mean community trophic levels.

A degraded reef fish community in Bocas del Toro is evidenced by low total biomass, under-representation of biomass at high trophic levels, and high abundance of small fishes, all classic symptoms of over-fishing (Pauly et al., 1998; Myers & Worm, 2003). Moreover, the range of total observed fish biomass represents the lowest value found amongst fish surveys conducted in the Caribbean. High-level carnivores and large fishes are depleted in intense fisheries (Cinner & McClanahan, 2006; Wilson et al., 2010), causing a skewing of the food web and community size structure. As described by Wilson et al. (2010), the loss of individuals within the largest size classes, which have the highest per capita reproductive output and produce the majority of juveniles, impacts the recruitment of small size classes of juvenile reef fish. Accordingly, we observed that small Haemulidae were rare on Bocas del Toro reefs. Exploitation thus appears to have contributed substantially to the distorted fish community patterns observed at Bocas del Toro (Guzmán et al., 2005; Cramer, 2013).

Another plausible hypothesis for the low total fish biomass and trophic shifts within the fish community in Bocas del Toro relative to other Caribbean sites is the loss of hard corals (Turner et al., 1999; Wilson et al., 2010). This in turn results in the loss of shelter and feeding grounds (Turner et al., 1999; Alevizon & Porter, 2015). This hypothesis was supported by significant negative correlations between the proportions of recently-dead corals and the biomass of fishes, as well as the finding that fish species that are known to associate with hard corals or hard substrate were rare. Moreover, fishes known to live on habitats of low complexity (particularly Pomacentridae and Gobiidae) and grazers (particularly Scaridae and Pomacentridae) occurred in very high abundances (Booth & Beretta, 1994; Bruggemann, Kuyper & Breeman, 1994).

Herbivores, detritivores and omnivores were overrepresented in the Bocas del Toro fish community compared to elsewhere in the Caribbean. Herbivorous species alone comprised nearly a third of the total fish biomass, which could be explained by a decreased number of predators in the system. Even though most herbivorous fishes were in the smallest size category (≤10 cm), this group has the potential to control the growth of macroalgae and prevent algal phase shifts, particularly in combination with invertebrate herbivores, such as sea urchins, which are abundant in this system (Kuempel & Altieri, 2017). However, if the reduction of live coral cover continues, herbivorous fishes may reach their limits for grazing control (Williams & Polunin, 2001; Williams, Polunin & Hendrick, 2001). Also, the lack of redundant species within the herbivore functional group is likely to result in low resilience, since a system with a single dominant herbivorous species (e.g., *Scarus iseri* with 72% of biomass in our system) is vulnerable to stressors affecting that species (Hughes, 1994; White & Jentsch, 2001). The reason for the dominance of one herbivore species may be attributable to the small body size of *S.iseri,* which matures at ∼65 mm. It is therefore not a targeted fishery species, and escapes most fishing pressure (Kuempel & Altieri, 2017).

Sponges cover up to 20% of substrata, and thus provide considerable physical structure on the Bocas del Toro reefs (Diaz & Rützler, 2001; Loh & Pawlik, 2014; Loh et al., 2015). In the absence of high cover of hard corals, sponges likely play an important role in supporting richness, biomass and expanded trophic levels of the depauperate fish community in our study system. We found evidence for such a positive effect, with an increased abundance of reef fishes with increased sponge cover. Sponges are major determinants of the rugosity and height of the reef (Diaz & Rützler, 2001), and thus could be an important driver for fish abundance and species richness in Bocas del Toro as in other Caribbean reef systems (Gratwicke & Speight, 2005). Sponges also comprise an important food source for spongivorous reef fishes, such as some members of Pomacentridae and Scarinae (Sammarco, Risk & Rose, 1987; Dunlap & Pawlik, 1996; Pawlik, 1998; Souza, Ilarri & Rosa, 2011). The pomacentrid *A. saxatilis* has been identified to have a functional dependency on sponges through either shelter or other aspects of habitat complexity that sponges provide (Gratwicke & Speight, 2005).

Proximity to mangroves was another important positive factor associated with fish communities, as the biomass and species richness of fishes were greater on coral reefs near mangroves. Mangroves are widely recognized for their functions of providing nursery grounds, shelter and food sources for reef fishes (Laegdsgaard & Johnson, 2001; Mumby et al., 2004). Our study suggests that the positive effect of mangroves as nursery and alternative adult habitats is an important factor maintaining diversity and biomass of the reef fish communities, and that this function remains particularly important in a system as degraded as Bocas del Toro. However, we did not find such evidence for seagrass meadows. Lowest fish species richness, biomass and trophic levels were found on reefs without mangroves in close proximity, presumably because many reef fish species depend on interconnectivity between habitat types (Ley, 2014).

Our findings suggest that Bocas del Toro, Panama is a model system for the study of reef fish communities that are associated with high levels of anthropogenic stress. Trends suggest that stressed systems are increasingly moving to low diversity, low mean trophic level, and a size distribution skewed to small body size (Pauly et al., 1998) as observed in Bocas del Toro. To maintain reef fish communities, resource managers should take factors such as sponge cover and proximity to other habitats, including mangroves, into consideration to prioritize protection efforts. Our results suggest that reef sponges and mangroves together can maintain physical structure, act as nurseries, and provide alternative habitats and thereby compensate for particular functional losses during coral mortality events. Much more information is nevertheless needed on the role of habitat connectivity if fisheries management is to be optimized and diversity hotspots safeguarded through effective spatial management that includes marine protected areas (Linton & Warner, 2003; Unsworth et al., 2008).

##  Supplemental Information

10.7717/peerj.4455/supp-1 Supplemental Information 1 Data for PCA Fish abundance, log transformed, fish > 12.5 excluded, factor analyses.

10.7717/peerj.4455/supp-2 Supplemental Information 2 Raw data for PCA, factor analyses

10.7717/peerj.4455/supp-3 Supplemental Information 3 Raw data for multivariate correlation

10.7717/peerj.4455/supp-4 Supplemental Information 4 Fish families by habitat

## Additional Information and Declarations

### Data Availability

The following information was supplied regarding data availability: The raw data have been provided in the Supplemental Files.

## References

[ref-1] Alevizon W, Porter J (2015). Coral loss and fish guild stability on a Caribbean coral reef: 1974–2000. Environmental Biology of Fishes.

[ref-2] Altieri AH, Harrison SB, Seemann J, Collin R, Diaz RJ, Knowlton N (2017). Tropical dead zones and mass mortalities on coral reefs. Proceedings of the National Academy of Sciences of the United States of America.

[ref-3] Alvarez-Filip L, Dulvy NK, Gill JA, Côté IM, Watkinson AR (2009). Flattening of Caribbean coral reefs: region-wide declines in architectural complexity. Proceedings of the Royal Society B: Biological Sciences.

[ref-4] Aronson RB, Bruno JF, Precht WF, Glynn PW, Harvell CD, Kaufman L, Rogers CS, Shinn EA, Valentine JF (2003). Causes of coral reef degradation. Science.

[ref-5] Aronson RB, Hilbun NL, Bianchi TS, Filley TR, Mckee BA (2014). Land use, water quality, and the history of coral assemblages at Bocas del Toro, Panamá. Marine Ecology Progress Series.

[ref-6] Beck MW, Heck Jr KL, Able KW, Childers DL, Eggleston DB, Gillanders BM, Halpern B, Hays CG, Hoshino K, Minello TJ (2001). The identification, conservation, and management of estuarine and marine nurseries for fish and invertebrates: a better understanding of the habitats that serve as nurseries for marine species and the factors that create site-specific variability in nursery quality will improve conservation and management of these areas. Bioscience.

[ref-7] Bell J, Galzin R (1984). Influence of live coral cover on coral-reef fish communities. Marine Ecology Progress Series.

[ref-8] Bellwood DR, Hughes TP, Folke C, Nystrom M (2004). Confronting the coral reef crisis. Nature.

[ref-9] Beulig A (1999). Relative influence of terrigenous vs. reef carbonate silt on turbidity and coral distribution at Bocas del Toro, Panama.

[ref-10] Booth DJ, Beretta GA (1994). Seasonal recruitment, habitat associations and survival of pomacentrid reef fish in the US Virgin Islands. Coral Reefs.

[ref-11] Bruggemann JH, Kuyper MWM, Breeman AM (1994). Comparative analysis of foraging and habitat use by the sympatric Caribbean parrotfish *Scarus vetula* and *Sparisoma viride* (Scaridae). Marine Ecology Progress Series.

[ref-12] Cesar HS (2000). Coral reefs: their functions, threats and economic value. Collected Essays on the Economics of Coral Reefs.

[ref-13] Cesar H, Burke L, Pet-Soede L (2003). The economics of worldwide coral reef degradation.

[ref-14] Cinner JE, McClanahan TR (2006). Socioeconomic factors that lead to overfishing in small-scale coral reef fisheries of Papua New Guinea. Environmental Conservation.

[ref-15] Collin R (2005). Ecological monitoring and biodiversity surveys at the Smithsonian Tropical Research Institute’s Bocas del Toro Research Station. Caribbean Journal of Science.

[ref-16] Cramer KL (2013). History of human occupation and environmental change in Western and Central Caribbean Panama. Bulletin of Marine Science.

[ref-17] Dahlgren CP, Kellison GT, Adams AJ, Gillanders BM, Kendall MS, Layman CA, Ley JA, Nagelkerken I, Serafy JE (2006). Marine nurseries and effective juvenile habitats concepts and applications. Marine Ecology Progress Series.

[ref-18] D’Croz L, Del Rosario JB, Gondola P (2005). The effect of fresh water runoff on the distribution of dissolved inorganic nutrients and plankton in the Bocas del Toro Archipelago, Caribbean Panama. Caribbean Journal of Science.

[ref-19] Diaz MC, Rützler K (2001). Sponges: an essential component of Caribbean coral reefs. Bulletin of Marine Science.

[ref-20] Dominici-Arosemena A, Wolff M (2005). Reef fish community structure in Bocas del Toro (Caribbean, Panama): gradients in habitat complexity and exposure. Caribbean Journal of Science.

[ref-21] Dunlap M, Pawlik JR (1996). Video-monitored predation by Caribbean reef fishes on an array of mangrove and reef sponges. Marine Biology.

[ref-22] Edgar GJ, Stuart-Smith RD (2014). Systematic global assessment of reef fish communities by the Reef Life Survey program. Scientific Data.

[ref-23] Edgar GJ, Stuart-Smith RD, Willis TJ, Kininmonth S, Baker SC, Banks S, Barrett NS, Becerro MA, Bernard ATF, Berkhout J, Buxton CD, Campbell SJ, Cooper AT, Davey M, Edgar SC, Forsterra G, Galvan DE, Irigoyen AJ, Kushner DJ, Moura R, Parnell PE, Shears NT, Soler G, Strain EMA, Thomson RJ (2014). Global conservation outcomes depend on marine protected areas with five key features. Nature.

[ref-24] Fabricius K, De’ath G, McCook L, Turak E, Williams DM (2005). Changes in algal, coral and fish assemblages along water quality gradients on the inshore Great Barrier Reef. Marine Pollution Bulletin.

[ref-25] Galzin R, Planes S, Dufour V, Salvat B (1994). Variation in diversity of coral reef fish between French Polynesian atolls. Coral Reefs.

[ref-26] Gardner TA, Côté IM, Gill JA, Grant A, Watkinson AR (2003). Long-term region-wide declines in Caribbean corals. Science.

[ref-27] Gratwicke B, Speight M (2005). The relationship between fish species richness, abundance and habitat complexity in a range of shallow tropical marine habitats. Journal of Fish Biology.

[ref-28] Guzmán HM, Barnes PAG, Lovelock CE, Feller IC (2005). A site description of the CARICOMP mangrove, seagrass and coral reef sites in Bocas del Toro, Panama Caribbean. Journal of Science.

[ref-29] Guzmán HM, Jiménez CE (1992). Contamination of coral reefs by heavy metals along the Caribbean coast of Central America (Costa Rica and Panama). Marine Pollution Bulletin.

[ref-30] Hixon MA, Johnson DW, Sogard SM (2014). BOFFFFs: on the importance of conserving old-growth age structure in fishery populations. ICES Journal of Marine Science.

[ref-31] Hodgson G (1999). A global assessment of human effects on coral reefs. Marine Pollution Bulletin.

[ref-32] Hughes TP (1994). Catastrophes, phase shifts, and large-scale degradation of a Caribbean coral reef. Science.

[ref-33] Jackson JBC, Kirby MX, Berger WH, Bjorndal KA, Botsford LW, Bourque BJ, Bradbury RH, Cooke R, Erlandson J, Estes JA, Hughes TP, Kidwell S, Lange CB, Lenihan HS, Pandolfi JM, Peterson CH, Steneck RS, Tegner MJ, Warner RR (2001). Historical overfishing and the recent collapse of coastal ecosystems. Science.

[ref-34] Jennings S, Lock JM, Polunin NVC, Roberts CM (1996). Population and ecosystem effects of reef fishing. Reef fisheries.

[ref-35] Kaufmann KW, Thompson RC (2005). Water temperature variation and the meteorological and hydrographic environment of Bocas del Toro, Panama. Caribbean Journal of Science.

[ref-36] Knowlton N, Brainard RE, Fisher R, Moews M, Plaisance L, Caley MJ, McIntyre A (2010). Coral reef biodiversity. Life in the world’s oceans: diversity distribution and abundance.

[ref-37] Kuempel CD, Altieri AH (2017). The emergent role of small-bodied herbivores in pre-empting phase shifts on degraded coral reefs. Scientific Reports.

[ref-38] Kuffner IB, Brock JC, Grober-Dunsmore R, Bonito VE, Hickey TD, Wright CW (2007). Relationships between reef fish communities and remotely sensed rugosity measurements in Biscayne National Park, Florida, USA. Environmental Biology of Fishes.

[ref-39] Laegdsgaard P, Johnson C (2001). Why do juvenile fish utilise mangrove habitats?. Journal of Experimental Marine Biology and Ecology.

[ref-40] Leinfelder RR, Seemann J, Heiss GA, Struck U (2012). Could ‘ecosystem atavisms’ help reefs to adapt to the Anthropocene?.

[ref-41] Ley JA, Stephen A. Bortone (2014). Mangrove connectivity helps sustain coral reef fisheries under global climate change. Interrelationships between corals and fisheries.

[ref-42] Linton DM, Warner GF (2003). Biological indicators in the Caribbean coastal zone and their role in integrated coastal management. Ocean & Coastal Management.

[ref-43] Loh T-L, McMurray SE, Henkel TP, Vicente J, Pawlik JR (2015). Indirect effects of overfishing on Caribbean reefs: sponges overgrow reef-building corals. PeerJ.

[ref-44] Loh T-L, Pawlik JR (2014). Chemical defenses and resource trade-offs structure sponge communities on Caribbean coral reefs. Proceedings of the National Academy of Sciences of the United States of America.

[ref-45] Mora C (2015). Ecology of fishes on coral reefs.

[ref-46] Mumby PJ, Edwards AJ, Arias-González JE, Lindeman KC, Blackwell PG, Gall A, Gorczynska MI, Harborne AR, Pescod CL, Renken H (2004). Mangroves enhance the biomass of coral reef fish communities in the Caribbean. Nature.

[ref-47] Myers RA, Worm B (2003). Rapid worldwide depletion of predatory fish communities. Nature.

[ref-48] Nagelkerken I, Roberts C, Van der Velde G, Dorenbosch M, Van Riel M, De La Moriniere EC, Nienhuis P (2002). How important are mangroves and seagrass beds for coral-reef fish? The nursery hypothesis tested on an island scale. Marine Ecology Progress Series.

[ref-49] Nagelkerken I, Van der Velde G, Gorissen MW, Meijer GJ, Van’t Hof T, Den Hartog C (2000). Importance of mangroves, seagrass beds and the shallow coral reef as a nursery for important coral reef fishes, using a visual census technique. Estuarine, Coastal and Shelf Science.

[ref-50] Paddack MJ, Reynolds JD, Aguilar C, Appeldoorn RS, Beets J, Burkett EW, Chittaro PM, Clarke K, Esteves R, Fonseca AC (2009). Recent region-wide declines in Caribbean reef fish abundance. Current Biology.

[ref-51] Pauly D, Christensen V, Dalsgaard J, Froese R, Torres F (1998). Fishing down marine food webs. Science.

[ref-52] Pawlik JR (1998). Coral reef sponges: do predatory fishes affect their distribution?. Limnology and Oceanography.

[ref-53] Riegl B, Purkis SJ, Keck J, Rowlands GP (2009). Monitored and modeled coral population dynamics and the refuge concept. Marine Pollution Bulletin.

[ref-54] Saila S, Kocic VL, McManus J (1993). Modelling the effects of destructive fishing practices on tropical coral reefs. Marine Ecology Progress Series.

[ref-55] Sammarco PW, Risk MJ, Rose C (1987). Effects of grazing and damselfish territoriality on internal bioerosion of dead corals: indirect effects. Journal of Experimental Marine Biology and Ecology.

[ref-56] Sammarco PW, Strychar KB (2009). Effects of climate change/global warming on coral reefs: adaptation/exaptation in corals, evolution in zooxanthellae, and biogeographic shifts. Environmental Bioindicators.

[ref-57] Seemann J (2013). The use of 13C and 15N isotope labeling techniques to assess heterotrophy of corals. Journal of Experimental Marine Biology and Ecology.

[ref-58] Seemann J, Gonzalez CT, Carballo-Bolaños R, Berry K, Heiss GA, Struck U, Leinfelder RR (2014). Assessing the ecological effects of human impacts on coral reefs in Bocas del Toro, Panama. Environmental Monitoring and Assessment.

[ref-59] Serafy JE, Shideler GS, Araújo RJ, Nagelkerken I (2015). Mangroves enhance reef fish abundance at the caribbean regional scale. PLOS ONE.

[ref-60] Souza A, Ilarri M, Rosa I (2011). Habitat use, feeding and territorial behavior of a Brazilian endemic damselfish Stegastes rocasensis (Actinopterygii: Pomacentridae). Environmental Biology of Fishes.

[ref-61] Spalding MD, Fox HE, Allen GR, Davidson N, Ferdaña ZA, Finlayson M, Halpern BS, Jorge MA, Lombana A, Lourie SA (2007). Marine ecoregions of the world: a bioregionalization of coastal and shelf areas. BioScience.

[ref-62] Stuart-Smith RD, Bates AE, Lefcheck JS, Duffy JE, Baker SC, Thomson RJ, Stuart-Smith JF, Hill NA, Kininmonth SJ, Airoldi L (2013). Integrating abundance and functional traits reveals new global hotspots of fish diversity. Nature.

[ref-63] Turner SJ, Thrush SF, Hewitt JE, Cummings VJ, Funnell G (1999). Fishing impacts and the degradation or loss of habitat structure. Fisheries Management and Ecology.

[ref-64] Unsworth RK, Salinas De Leon P, Garrard SL, Jompa J, Smith DJ, Bell JJ (2008). High connectivity of Indo-Pacific seagrass fish assemblages with mangrove and coral reef habitats. Marine Ecology-Progress Series.

[ref-65] White PS, Jentsch A, Esser K, Lüttge U, Kadereit JW, Beyschlag W (2001). The search for generality in studies of disturbance and ecosystem dynamics. Progress in botany.

[ref-66] Williams I, Polunin N (2001). Large-scale associations between macroalgal cover and grazer biomass on mid-depth reefs in the Caribbean. Coral Reefs.

[ref-67] Williams ID, Polunin NVC, Hendrick VJ (2001). Limits to grazing by herbivorous fishes and the impact of low coral cover on macroalgal abundance on a coral reef in Belize. Marine Ecology Progress Series.

[ref-68] Wilson SK (2006). Multiple disturbances and the global degradation of coral reefs: are reef fishes at risk or resilient?. Global Change Biology.

[ref-69] Wilson SK, Fisher R, Pratchett MS, Graham NAJ, Dulvy NK, Turner RA, Cakacaka A, Polunin NVC (2010). Habitat degradation and fishing effects on the size structure of coral reef fish communities. Ecological Applications.

[ref-70] Zaneveld JR, Burkepile DE, Shantz AA, Pritchard CE, McMinds R, Payet JP, Welsh R, Correa AMS, Lemoine NP, Rosales S, Fuchs C, Maynard JA, Thurber RV (2016). Overfishing and nutrient pollution interact with temperature to disrupt coral reefs down to microbial scales. Nature Communications.

