# Supplementary figures and images for: The importance of sponges and mangroves in supporting fish communities on degraded coral reefs in Caribbean Panama
Source: PeerJ. 2018 Mar 29;6:e4455. doi: 10.7717/peerj.4455 (PMC5878927; doi:10.7717/peerj.4455)

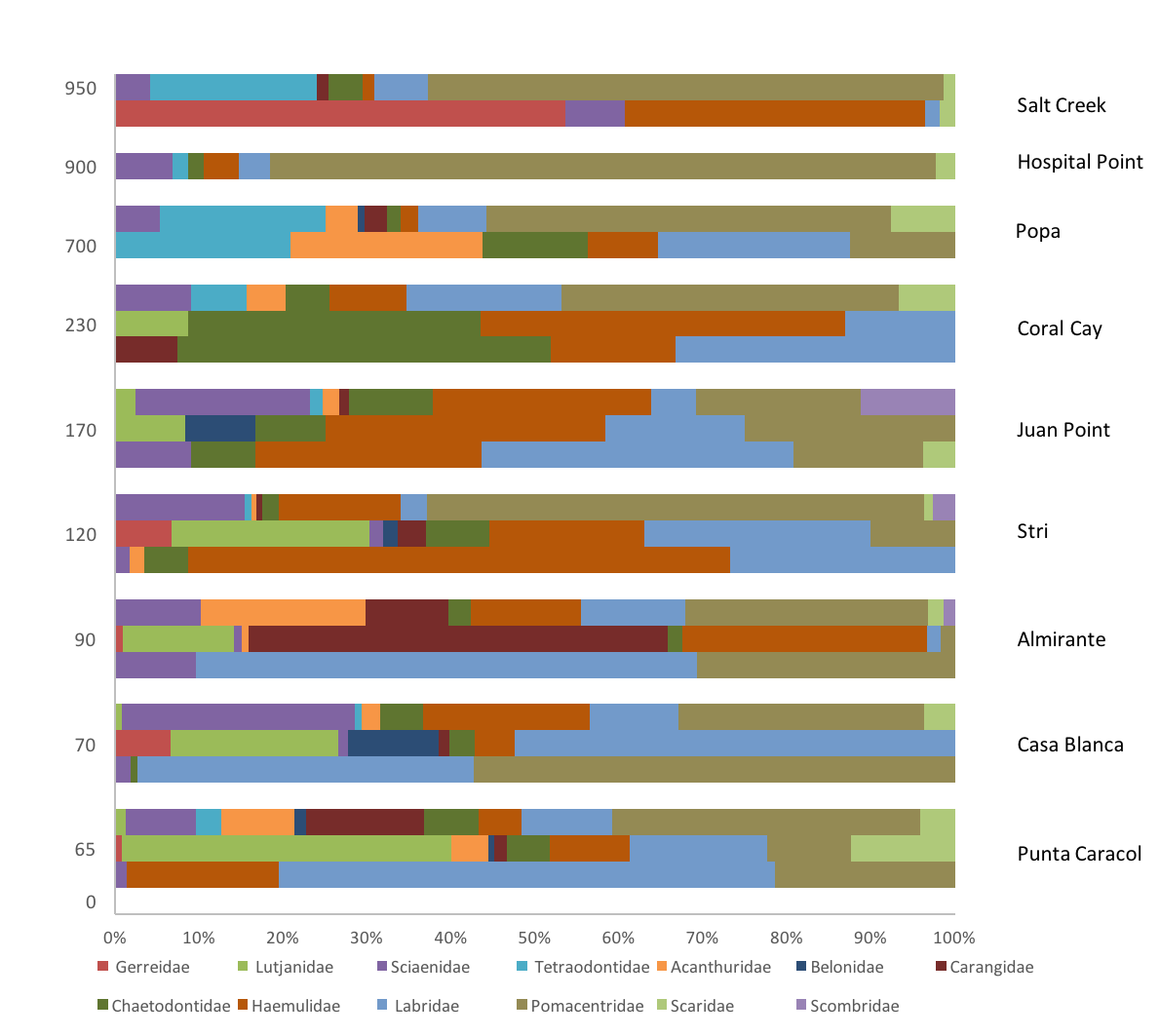

Supplement: Supplemental Information 4 [file peerj-06-4455-s004.png]
